# Synthesis and Bioactivity Characterization of Scutellarein Sulfonated Derivative

**DOI:** 10.3390/molecules22061028

**Published:** 2017-06-21

**Authors:** Ting Gu, Yue Zhong, Yu-Ting Lu, Ying Sun, Ze-Xi Dong, Wen-Yu Wu, Zhi-Hao Shi, Nian-Guang Li, Xin Xue, Fang Fang, He-Min Li, Yu-Ping Tang

**Affiliations:** 1 National and Local Collaborative Engineering Center of Chinese Medicinal Resources Industrialization and Formulae Innovative Medicine, Nanjing University of Chinese Medicine, Nanjing 210023, Jiangsu, China; guting1992@163.com (T.G.); 18260028701@163.com (Y.Z.); lyt1996tian@outlook.com (Y.-T.L.); 18260028601@163.com (Y.S.); dongzexi1215@163.com (Z.-X.D.); 15150513147@163.com (W.-Y.W.); xuexin0540121@163.com (X.X.); fylffh@163.com (F.F.); lihemin2006@sina.com (H.-M.L.); 2 Department of Medicinal Chemistry, Nanjing University of Chinese Medicine, Nanjing 210023, Jiangsu, China; 3 Department of Organic Chemistry, China Pharmaceutical University, Nanjing 211198, Jiangsu, China; sszh163@163.com

**Keywords:** scutellarin, scutellarein, sulfonated derivative, solubility, antioxidant, antithrombic

## Abstract

Scutellarin (**1**) has been widely used to treat acute cerebral infarction in clinic, but poor aqueous solubility decreases its bioavailability. Interestingly, scutellarin (**1**) could be metabolized into scutellarein (**2**) in vivo. In this study, a sulfonic group was introduced at position C-8 of scutellarein (**2**) to enhance the aqueous solubility of the obtained derivative (**3**). DPPH (1,1-diphenyl-2-picrylhydrazyl)-radical scavenging ability and antithrombic activity were also conducted to determine its bioactivity. The result showed that scutellarein derivate (**3**) could be a better agent for ischemic cerebrovascular disease treatment.

## 1. Introduction

Ischemic cerebrovascular disease is a common and high-incidence disease that threatens human health seriously by severe neuronal injury and loss of brain function [1]. Stroke is a typical symptom of ischemic cerebrovascular disease, and causes 9% of all deaths worldwide [2]. Thus, serious harm of individual’s physical, mental, and social functioning can occur as a result of ischemic cerebrovascular diseases [3,4].

*Erigeron breviscapus* (Vant.) Hand-Mazz is one of the well-known Chinese herbs, and has an extensive application in traditional Chinese medicine treatment. The mechanism of *E. breviscapus* has aroused increasing attention because of its significant curative effect. Liu studied the antioxidant activity of the ethanol extract of *E. breviscapus*, and revealed it played an important role in neuroprotective actions [5]. Tao also found that *E. breviscapus* ethanol extract had therapeutic applications in neurological diseases due to the inhibition of γ-aminobutyric acid transaminase (GABA-T) and succinic semialdehyde dehydrogenase (SSADH) [6].

**Figure 1:**
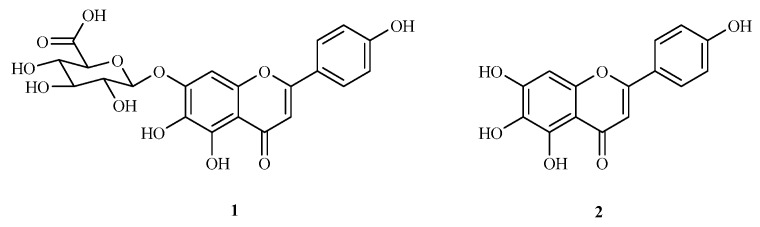
Structures of scutellarin (**1**) and scutellarein (**2**).

By modern analysis methods, scutellarin (4′,5,6-trihydroxyflavone-7-glucuronide) (**1**) (Figure 1) could be confirmed as an important active ingredient in *Erigeron breviscapus* (Vant.) Hand-Mazz [7,8]. Ke found that scutellarin could inhibit hepatocellular carcinoma (HCC) cell metastasis in vivo, and migration and invasion in vitro by downregulating the STAT3/Girdin/Akt signaling [9]. Further, in Li’s research, scutellarin could attenuate vasospasm and neurological deficits by modulating the Erk5-KLF2-eNOS pathway after subarachnoid hemorrhage (SAH) [10]. Furthermore, Zhao found that scutellarin was a promising therapeutic agent for the prevention of wear particle-induced periprosthetic osteolysis [11]. In addition, scutellarin (**1**) is effective in treating cerebral ischemia, angina pectoris, myocardial infarction, stroke, cerebral thrombotic diseases and other kinds of cardiovascular diseases and cerebrovascular injury [12].

However, because of the poor aqueous solubility (14.4 ± 0.17 μg/mL) [13], and the absolute oral low bioavailability in Beagle dog (0.4%) [14], there is not a wide range of applications of scutellarin (**1**) in clinic. Structure modification and dosage form transformation are two common methods used to increase the bioavailability of this kind of natural product. By the approaches of being bound to polyrotaxane (PR) [15], adopting different lipid excipients such as cremophor EL [16], or synthesizing glucose-containing scutellarein derivatives [17], the bioavailability of scutellarin (**1**) can be improved to some extent.

Scutellarein (**2**) (Figure 1) is the hydrolysate of scutellarin (**1**), and is the main absorbing form in vivo [18]. Our previous work indicated that scutellarein (**2**) displayed better protective effect in rat cerebral ischemia than scutellarin (**1**) [19,20]. Nevertheless, the problem of poor aqueous solubility is still not resolved due to the flavonoid skeleton of scutellarein (**2**).

Sulfonation is an important reaction in increasing the solubility of flavonoids while retaining the chemical properties of the parent compounds [21]; as a well-known type of in vivo metabolic conversion, it also enhances the aqueous solubility of the ingested compounds [22]. Quercetin is a typical flavonoid with an antiproliferative effect on a wide range of cancer cells [23,24,25]. Cui, Woźnicka, and Zhang introduced sulfonic groups on quercetin [21,22,26], and all of these quercetin derivatives showed good aqueous solubility on the basis of bioactivity preservation. 

Therefore, in this research, we synthesized the sulfonated derivative of scutellarein (**2**), and a series of biological activity assays including aqueous solubility, (1,1-diphenyl-2-picrylhydrazyl) (DPPH)-radical scavenging ability, and antithrombic activity were also conducted to study its biological activity.

## 2. Results and Discussion

### 2.1. Chemical Synthesis

**Scheme 1:**
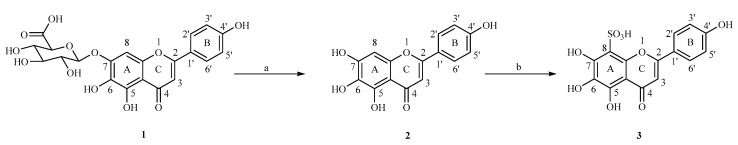
The route of the synthesis of scutellarein derivate (**3**). (**a**) H_2_SO_4_, EtOH, 120 °C, 48 h, 17.3%; (**b**) H_2_SO_4_, 25 °C, 3 h, 18%.

In this study, one of our previous methods was applied to synthesize scutellarein (**2**) by the hydrolysis of scutellarin (**1**) [19]. In this method, 3.0 mol/L H_2_SO_4_ in 90% ethanol and heating under a N_2_ atmosphere at 120 °C for 48 h were selected as the best condition for the synthesis of scutellarein (**2**), and the yield could be up to 17.3%. Subsequently, concentrated sulfuric acid—as reactant and solvent—was adopted to introduce a sulfonic group at the C-8 position of the scutellarein (**2**). The reaction temperature should be controlled at about 25 °C to avoid the side reaction in B ring. According to the ^1^HNMR spectrum of **3**, the coupling constants of C2′,6′-H and C3′,5′-H were both 8.7, which indicated that the sulfonic group was not at C2′,6′ and C3′,5′ positions. Furthermore, the hydrogen signal at δ 6.72 of C3-H was still present; this information confirmed that the sulfonic group was introduced at the C-8 position. The synthesis route is shown in Scheme 1.

### 2.2. Aqueous Solubility

The aqueous solubility of scutellarin (**1**), scutellarein (**2**), and **3** were determined by UV spectrophotometry [27,28,29]. As presented in Table 1, the aqueous solubility of scutellarin (**1**), scutellarein (**2**), and **3** were 7.62, 6.85, and 1949.64 μg/mL, respectively. The results showed that the introduction of the sulfonic group increased its aqueous solubility significantly compared to scutellarin (**1**) and scutellarein (**2**).

### 2.3. DPPH-Radical Scavenging Ability Assay

Antioxidants play important roles in removing free radicals, as antioxidants provide hydrogen to free radicals and prohibit the adverse reactions from destroying normal tissues. This assay was measured according to previous studies [30]. The results are displayed in Table 1.

As shown in the results, scutellarin (**1**), scutellarein (**2**), and **3** all exhibited good DPPH-radical scavenging ability in this assay, as the values of IC_50_ were 17.88, 16.05, and 16.78 µM, respectively, which indicated that compound **3** still had antioxidant activity.

### 2.4. Antithrombic Assay

According to our previous studies, the antithrombotic activity could be estimated by measuring the prolongation of the plasma clotting time of thrombin time (TT), activated partial thromboplastin time (APTT), international normalized ratio (INR) increase in prothrombin time (PT), and reduction in fibrinogen (FIB) content [31]. The results are displayed in Table 2.

As the results show, the antithrombotic activity was remained when the glucuronyl group in scutellarin (**1**) was hydrolyzed to produce scutellarein (**2**); although the PT decreased and the FIB content increased in scutellarein (**2**), the TT and APTT in scutellarein (**2**) increased compared with those in scutellarin (**1**); this result indicated that the glucuronyl group was not important for the antithrombotic activity.

The results also indicated that scutellarein derivative with a sulfonic group at the C-8 position (**3**) could retain the antithrombotic activity. On the one hand, the introduction of a sulfonic group at the C-8 position increased TT and PT significantly compared with the control group. Specifically, the TT prolongation of scutellarein derivative (**3**) (23.71 s) was better than scutellarin (**1**) (23.25 s), but inferior to scutellarein (**2**) (24.48 s); nevertheless, the PT prolongation of scutellarein derivative (**3**) (6.33 s) was superior to scutellarin (**1**) (6.25 s) and scutellarein (**2**) (5.93 s). On the other hand, the introduction of a sulfonic group at the C-8 position of scutellarein (**3**) increased APTT and decreased FIB to some degree in comparison to the control group; however, the improvement of APTT (30.33 s) and FIB (7.01 g/L) was not as good as scutellarin (**1**) (APTT: 33.78 s, FIB: 6.41 g/L) and scutellarein (**2**) (APTT: 36.12 s, FIB: 6.91 g/L).

Thus, the introduction of sulfonic group at C-8 position of the scutellarein (**3**) could retain the antithrombotic activity of the parent compounds.

## 3. Experimental Section

### 3.1. General Information

Scutellarin (**1**) was purchased from Sichuan Mianning Jiexiang Materials Co. Ltd. (Chengdu, China). Reagents and solvents were purchased from commercial sources and used without further purification unless otherwise specified. All non-aqueous reactions were carried out under nitrogen production using flame-dried glassware; the anhydrous solvents were transferred via syringe or stainless steel cannula. Organic solvents were concentrated below 45 °C by Büchi rotary evaporator at approximately 20 mm Hg. Then, 0.15–0.20 mm silica gel plates (RSGF 254, Yantai, China), as thin-layer chromatography (TLC), was used to monitored all of the reactions in 254 nm UV light. Chromatography was carried out on silica gel (160–200 mesh, Qingdao, China) with mixtures of petroleum ether (60–90) and ethyl acetate as eluant. The ^1^H NMR spectroscopy was carried out on a Bruker AV-300 (300 MHz) (Bruker Corporation, Karlsruhe, Germany) in DMSO-*d*_6_. Abbreviations used are s (singlet), d (doublet), t (triplet), q (quartet), b (broad), and m (multiplet). Mass spectrometry (MS) were performed on a Waters Synapt HDMS spectrometer equipped with an electrospray ionization source (ESI).

### 3.2. Synthesis

#### 3.2.1. 5,6,7-Trihydroxy-2-(4-hydroxyphenyl)-4*H*-chromen-4-one (**2**)

Concentrated H_2_SO_4_ (100 mL) was added into a stirring mixture of **1** (10 g, 21.65 mmol) and water (10 mL) in ethanol (90 mL), and the reaction mixture was refluxed for 48 h under nitrogen and then was allowed to cool to room temperature. Water (1000 mL) was added, and the solid that appeared was filtered and then purified by column chromatography on silica gel with 50% ethyl acetate in petroleum ether as eluent to afford **1** (1.05 g, 17% yield) as a yellow solid. IR (KBr): 3343.24, 1663.08, 1610.20, 1580.85, 1458.41, 1364.24, 1270.26, 1252.35, 1183.51, 1079.25, 1028.97, 1004.24, 830.86, 721.26, 598.71, 574.65 cm^−1^. ^1^H-NMR (DMSO-*d*_6_) 6.73 (s, 1H, 8-H), δ 6.78 (s, 1H, 3-H), 6.90–6.93 (d, 2H, *J* = 8.8 Hz, 3′,5′-H), 7.90–7.93 (d, 2H, *J* = 8.8 Hz, 2′,6′-H), 8.71 (s, 1H, 6-OH), 10.30 (s, 1H, 4′-OH), 10.44 (s, 1H, 7-OH), 12.79 (s, 1H, 5-OH); ESI-MS: *m/z* 285 [M − H]^−^; Anal. Calcd. for C_15_H_10_O_6_: C, 62.94; H, 3.52; Found: C, 62.92; H, 3.49. The IR and ^1^H-NMR spectrums were included in the Supplementary Materials available online.

#### 3.2.2. 5,6,7-Trihydroxy-2-(4-hydroxyphenyl)-4-oxo-4*H*-chromene-8-sulfonic acid (**3**)

Concentrated sulfuric acid (15 mL) was added to **2** (1.0 g) in a 50 mL round-bottom flask, the reaction mixture was allowed to continue stirring for 12 h at 25 °C. After that, 25 mL of saturated sodium chloride solution was added into the mixture, and the mixture was kept for 4 h before filtering. The orange–red precipitate was washed with saturated sodium chloride solution until the pH value of the filtrate was up to 7, and was then recrystallized twice from the hot saturated aqueous solution; **3** (230 mg, 18% yield) was obtained as yellow solid. IR (KBr): 3272.90, 2918.66, 1659.91, 1592.18, 1558.24, 1472.13, 1417.77, 1373.40, 1321.34, 1259.45, 1229.59, 1163.55, 1105.26, 1025.93, 672.43, 579.93, 522.46 cm^−1^. ^1^H-NMR (DMSO-*d*_6_) δ 6.72 (1H, s, 3-H), 6.97–6.99 (2H, d, *J* = 8.7 Hz, 3′,5′-H), 8.27–8.30 (2H, d, *J* = 8.7 Hz, 2′,6′-H), 8.55 (1H, s, 6-OH), 11.13 (1H, s, 4-OH), 12.52 (1H, s, 7-OH), 12.99 (1H, s, 5-OH); ESI-MS: *m/z* 365 [M − H]^−^; Anal. Calcd. for C_15_H_10_O_9_S: C, 49.19; H, 2.75; S, 8.75; Found: C, 49.21; H, 2.78; S. 8.77. The IR and ^1^H-NMR spectrums were included in the Supplementary Materials available online.

### 3.3. Aqueous Solubility

The solubility of scutellarin (**1**), scutellarein (**2**), and **3** in water was determined using the known method [27,28,29]. According to multicomponent exploitation method, 334 nm could be considered as the wavelength of maximum absorbance of scutellarein (**2**) by UV absorption spectrophotometry. Scutellarin (**1**) and scutellarein (**2**) (300 μg) were dissolved in 25 mL CH_3_OH, and **3** (300 μg) was dissolved in 25 mL water. The solutions of these three compounds had concentrations ranging from 3 to 12 μg/mL. Standard curves were determined on the basis of the absorbances of test samples obtained by UV scanning, and all of them showed a good linear relationship. Each tested compound (250 μg) was ultrasound dissolved in 10 mL pure water for 1 h at 25 °C, and the solutions were stood for 30 min before centrifuging at the speed of 30,000 r/min. Absorbances of each compound were obtained by UV scanning, and the aqueous solubility of all three compounds were obtained through analysis of the standard curve.

### 3.4. DPPH-Radical Scavenging Ability Assay

The following method was adopted to evaluate the 1,1-diphenyl-2-picrylhydrazyl (DPPH) scavenging property of the products: scutellarin (**1**), scutellarein (**2**), and **3** were dissolved in DMSO to result in final concentrations ranging from 16 to 250 μmol/L, and then DPPH (80 μmol/L, 100 μL) dissolved in DMSO was added in the testing samples. After incubating for 30 min in the dark, the scavenging effect was calculated according to the following equation:

Scavenging effect (%) = [(1 − (A_1_ − A_2_)/A_0_] × 100% (1)

where A_0_ is the absorbance of the control (without extract), A_1_ is the absorbance in the presence of the extract, and A_2_ is the absorbance without DPPH.

### 3.5. Antithrombic Assay

Male New Zealand white rabbits weighing 2–2.5 kg were obtained from the experimental animal center of Nanjing University of Chinese Medicine and were approved by Animal Ethics Committee of Nanjing University of Chinese Medicine. They were kept in plastic cages at 22 ± 2 °C with free access to pellet food and water and on a 12 h light/dark cycle. Animal welfare and experimental procedures were carried out in accordance with the guide for the care and use of laboratory animals (National Research Council of USA, 1996) and related ethical regulations of Nanjing University of Chinese Medicine. Rabbits were anesthetized with pentobarbital (50 mg/kg) and blood was drawn from the common carotid artery. Blood was collected into plastic tubes with 3.8% sodium citrate (citrate/blood: 1:9, *v/v*) for plasma anticoagulation. Platelet-poor plasma (PPP) was separated from blood by centrifugation at 3000 rpm for 10 min.

TT, APTT, PT, and FIB were tested with commercial kits following the manufacturer’s instructions by a coagulometer (Model LG-PABER-I, Steellex Co., Beijing, China). All the compounds were dissolved in PBS with 20% DMSO, and the concentration was 100 μM. TT was determined by incubating 40 μL PPP solution for 3 min at 37 °C, followed by addition of 40 μL thrombin solution and 20 μL sample for 3 min at 37 °C. APTT was determined by incubating 10 μL sample solution and 50 μL PPP solution with 50 μL APTT-activating agent for 3 min at 37 °C, followed by the addition of 50 μL CaCl_2_. PT was determined by incubating 40 μL PPP solution for 3 min at 37 °C, followed by the addition of 40 μL thromboplastin agent and 20 μL sample. FIB was determined by incubating 10 μL PPP solution with 90 μL imidazole buffer for 3 min at 37 °C, followed by addition of 50 μL FIB agent and 10 μL sample solution. By measuring the prolongation of the plasma clotting time of thrombin time (TT), activated partial thromboplastin time (APTT), INR increase in prothrombin time (PT), and reduction in fibrinogen (FIB) content, the antithrombotic activity was assessed.

## 4. Conclusions

Scutellarin (**1**) is effective in treating cardiovascular diseases and cerebrovascular injury [12]; nevertheless, clinical application is limited because of its poor solubility and low bioavailability. In this study, we introduced a sulfonic group at the C-8 position of scutellarein (**2**), which increased the aqueous solubility obviously. Further, DPPH-radical scavenging ability assay and antithrombic assay also proved that the sulfonated derivative (**3**) still retained the bioactivity of parent compounds. This kind of scutellarein derivative (**3**) could be an efficient agent for ischemic cerebrovascular disease treatment.

## Figures and Tables

**Table 1:** The solubility in water and antioxidant activity of scutellarin (**1**), scutellarein (**2**), and **3**. DPPH: (1,1-diphenyl-2-picrylhydrazyl).

Compd.	Solubility (μg/mL)	DPPH (μM)
Scutellarin (**1**)	7.62	17.88
Scutellarein (**2**)	6.85	16.05
(**3**)	1949.64	16.78

**Table 2:** The thrombin inhibition activity of scutellarin (**1**), scutellarein (**2**), and **3**. APTT: activated partial thromboplastin time; FIB: fibrinogen; PT: prothrombin time; TT: thrombin time.

Compd.	Plasma Coagulation Parameters			
	TT (s)	APTT (s)	PT (s)	FIB (g/L)
Blank plasma	19.85 ± 1.59	29.83 ± 3.86	5.03 ± 0.24	7.23 ± 0.38
Scutellarin (**1**)	23.25 ± 1.55	33.78 ± 2.32	6.25 ± 0.08	6.41 ± 0.15
Scutellarein (**2**)	24.48 ± 1.18	36.12 ± 2.20	5.93 ± 0.81	6.91 ± 0.12
**3**	23.71 ± 1.82	30.33 ± 1.06	6.33 ± 1.87	7.01 ± 0.96

Data represent mean ± SD *n* = 4.

## Supplementary Materials

The following are available online: IR and ^1^H-NMR spectrums of **2** and **3**.
