# Supplementary material for: Synthesis and Bioactivity Characterization of Scutellarein Sulfonated Derivative
Source: Molecules. 2017 Jun 21;22(6):1028. doi: 10.3390/molecules22061028 (PMC6152701; doi:10.3390/molecules22061028)

5,6,7-Trihydroxy-2-(4-hydroxyphenyl)-4*H*-chromen-4-one (**2**)

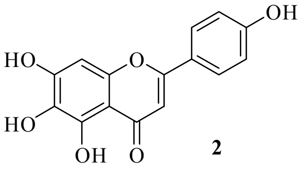

2

^{1}H-NMR of **2**

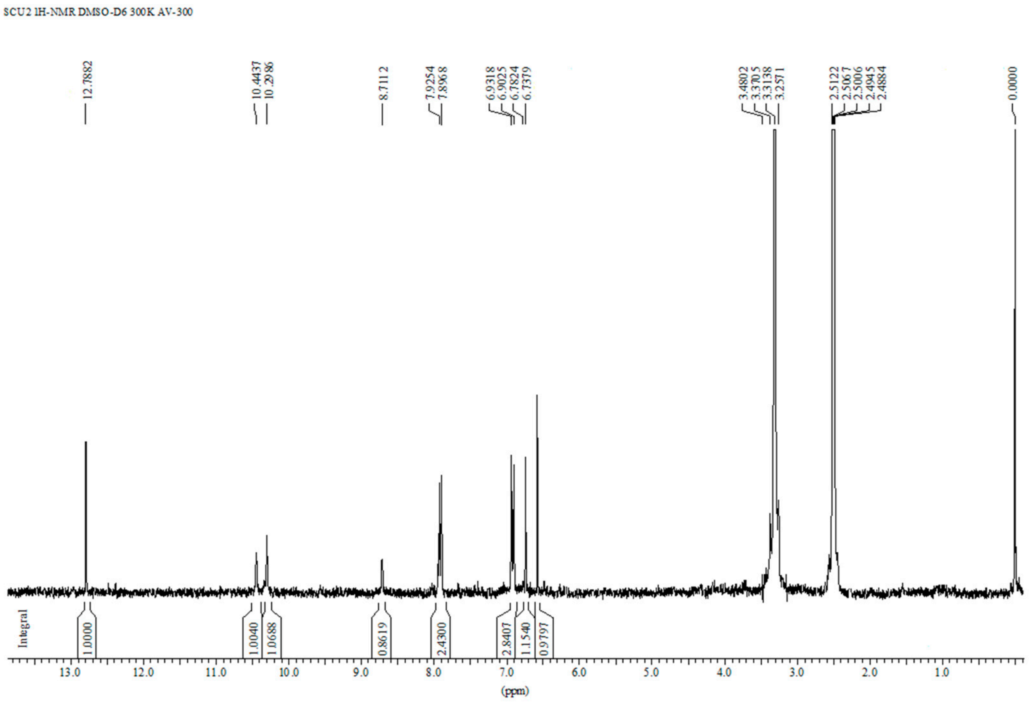

IR of **2**

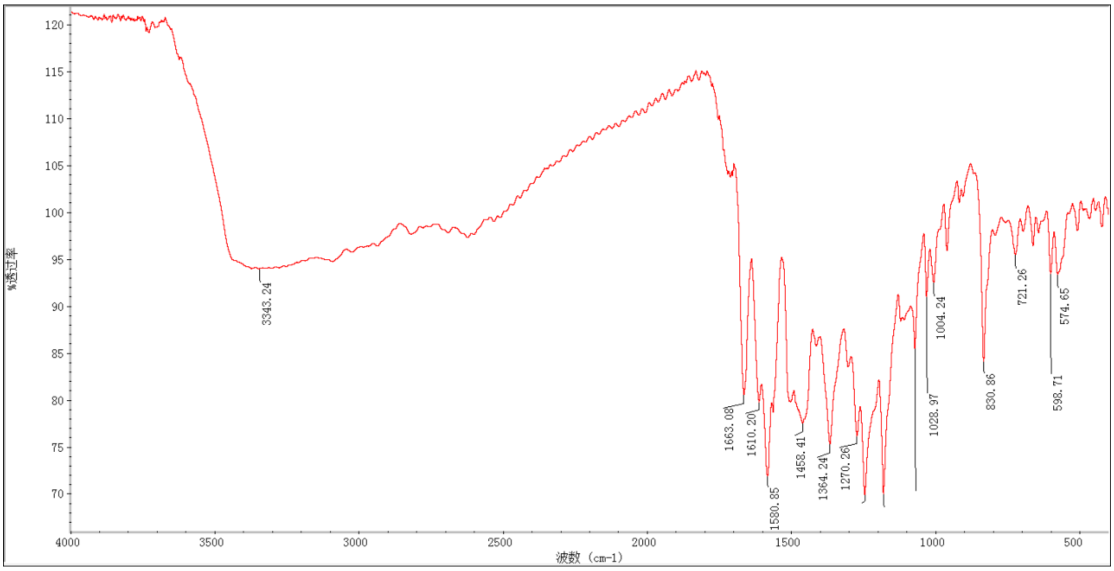

5,6,7-Trihydroxy-2-(4-hydroxyphenyl)-4-oxo-4*H*-chromene-8-sulfonic acid (**3**)

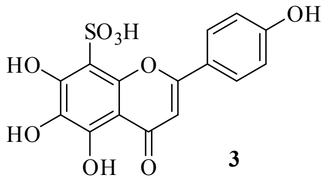

3

^{1}H-NMR of **3**

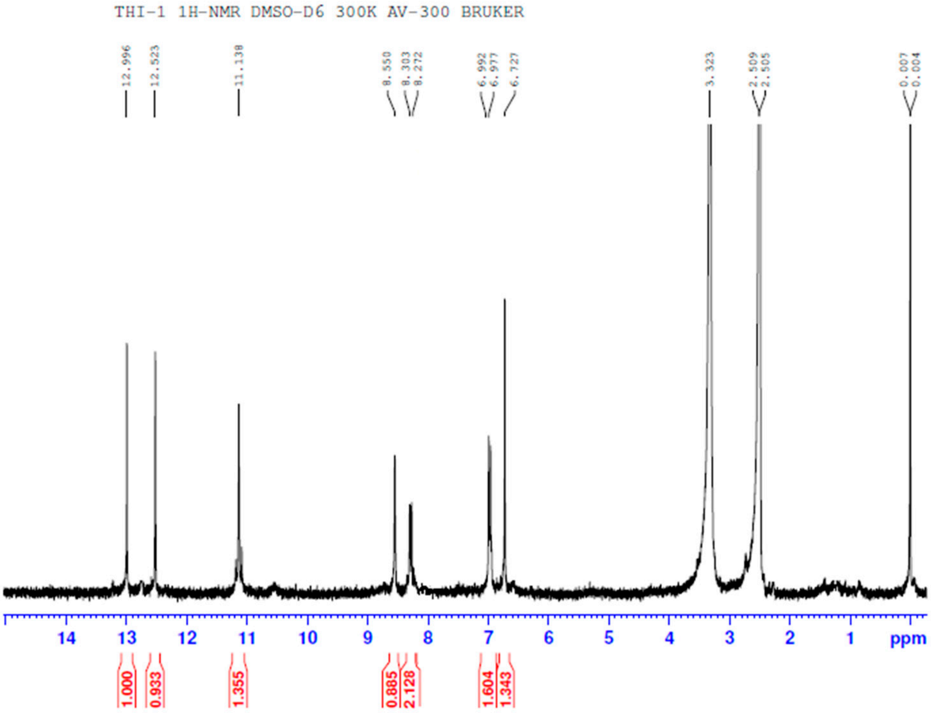

IR of **3**

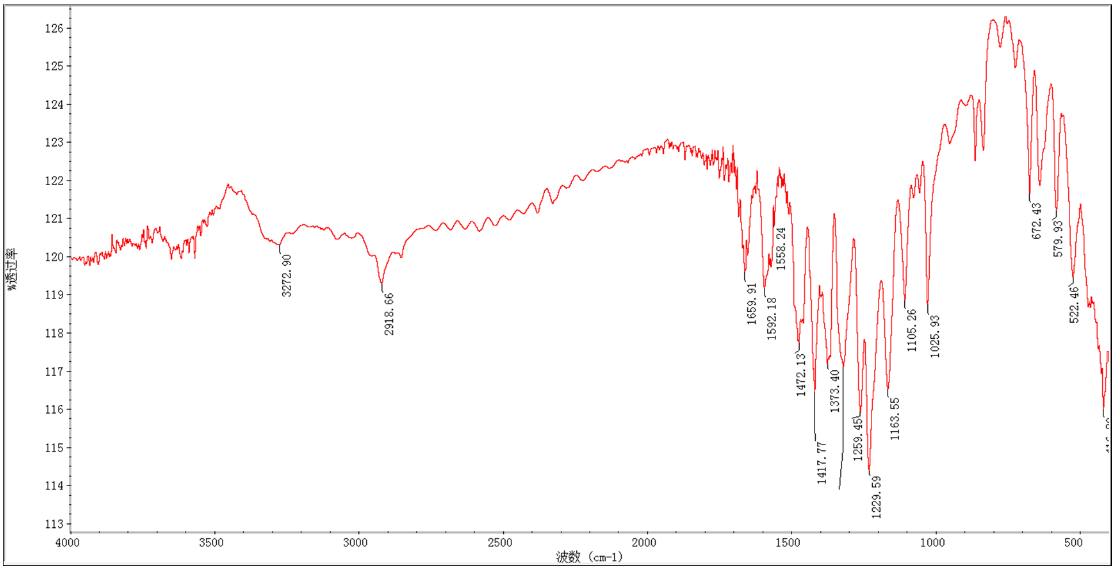

Supplement: Supplementary file 1 [file molecules-22-01028-s001.pdf]
